# Evaluation of a Mentor training program for midwives in two hospitals in Warsaw, Poland - a qualitative descriptive study

**DOI:** 10.1186/s12909-021-02769-7

**Published:** 2021-06-15

**Authors:** Małgorzata Stefaniak, Ewa Dmoch-Gajzlerska

**Affiliations:** Department of Obstetrics and Gynecology Didactics, Faculty of Health Sciences, Medical University of Warsaw, ul. Litewska 14/16, 00-575 Warsaw, Poland

**Keywords:** Mentoring training, Mentor, Midwife, Education, Midwifery

## Abstract

**Background:**

Mentoring is a recognized, but still underutilized strategy for effective clinical training of midwifery students. The success of formally recognized course-embedded mentoring depends on adequate preparation of clinical teacher to act as mentors and effective developing of their mentoring skills. The aim of this study was to evaluate a Mentor Training Program for midwives, the first of its kind in Poland.

**Methods:**

Twenty-one midwives who completed a dedicated Mentor Training Program organized by the Medical University of Warsaw (12–14 June 2017) participated in this study. In Stage 1, 7 days prior to the training course, the participants completed an online questionnaire that identified their motivations and expectations. In Stage 2, they assessed the training program they had completed as well as described any perceived needs of further mentor training. Subsequently a qualitative study of the data was performed using content analysis.

**Results:**

In Stage 1, the participants expressed their interest in the Mentor Training Program mostly expecting to develop skills allowing them to implement mentoring in clinical training of student midwives. They were aware of the potential benefits of mentoring for hands-on instruction on the wards and wanted to gain knowledge of this strategy. In Stage 2, the overall satisfaction with the program was high, but the participants assessed their preparedness to act as midwifery mentors as inadequate. The results suggest that the Mentor Training Program should be further refined and expanded while the outcomes need to be evaluated in more detail by both new mentors and their trainers over a longer period of time, possibly after the participants have gained actual experience of mentoring in the clinical setting.

**Conclusion:**

The participants described the Mentor Training Program as innovative, valuable and largely meeting their expectations. They gained knowledge of the concept of mentoring and its potential application in clinical training of midwifery students in Poland. Future mentor training programs should be expanded with a greater focus on developing, strengthening and applying mentoring skills in the clinical setting.

**Supplementary Information:**

The online version contains supplementary material available at 10.1186/s12909-021-02769-7.

## Background

The optimal preparation of graduates to deliver high-quality care in different healthcare settings is an obvious key target for medical universities which offer degree courses in midwifery [1–4]. In accordance with the European Union (EU) requirements midwifery training must be given on a full time basis and comprise at least 3 years or 4720 h while practical clinical training must constitute one half of the training [5]. Learning from experience with emphasis on a hands-on approach is therefore seen as a crucial component of effective preparation for the future work on the wards. The experiential learning cycle was described by David Kolb in his model published in 1984. Experiential learning is basically the transformation of experience to knowledge. Learning orientations could be described as tensions between active experimentation vs. reflective observation and abstract conceptualization vs. concrete experience [6]. In the context of clinical training in midwifery these are most effectively achieved through bedside training which entails reflective observation and actual experience of patient care. Another education theory to be considered for incorporation in the development of clinical placements for student midwives is the competency-based education (CBE). It is a model adopted by many academic institutions because it links theory to practice [7–9]. CBE has been defined in many ways and interpreted differently across academic curricula. The theoretical foundation of CBE has multiple learning theory roots: behaviorist, functionalist, and humanistic learning theories. In the context of medical education, including midwifery, the important feature of the competency-based approach is that it integrates the knowledge, skills and attitudes (behavior). Importantly, it is necessary for students to learn the theoretical foundations to best understand how to apply their learning in practice [9].

Better access to information technology and digital resources as well as new communication channels and virtual learning communities have offered a chance of new approaches to learning. One of these approaches is self-regulated learning [10]. According to Brockett et al. the term refers to both the external characteristics of an instructional process and the internal characteristics of the learner, where the individual assumes primary responsibility for a learning experience [11]. Within cognitive psychology, self-regulated learning has been considered students’ independence in learning. Self-regulated learning is an active, constructive process whereby learners set goals for their learning and attempt to monitor, regulate and control their cognition, motivation, and behavior, guided and constrained by their goals and contextual features of the environment [10]. Obviously, self-regulated learning is to some extent used in midwifery education but alone it would not provide student midwives with knowledge and skills for effective maternity care. To be effective and safe practical clinical training must be guided and supervised by an experienced practitioner. Training on the wards student midwives need a safe and supportive learning environment [12] to understand how to apply their learning in practice. A solution is offered by a mentor-led competency-based clinical training whereby midwifery mentors aid midwifery students (mentees) in attaining the competencies identified in the curriculum, diagnose mentee needs, steer them in the right direction, offer support through the learning process until mentees have attained the identified competencies, and are co-accountable for the training process [9]. This approach offers a chance of a better preparation of students for their work in the future [3]. Having an assigned, dedicated mentor helps students to adapt to the new setting, to build self-confidence and to expand their theoretical knowledge and practical skills guided by an experienced practitioner. The research confirms that a CBE model includes an emphasis on outcomes, a strong pedagogy, the use of interdisciplinary resources, and assessment of a student attainment of competencies across the curriculum [8, 9]. A student demonstrates readiness to graduate when they can demonstrate the knowledge, skills, attitudes, values, and behavior gained through attainment of the identified competencies [9]. Individual teaching is offered to those students who have problems in the attainment of the competencies [13]. Book et al. outline a process by which competencies measure student learning by specifically stating the levels of performance a student is expected to master across the curriculum [13]. Gervais argue that competencies are different from goals and learning objectives. Competencies are written in the present tense and based on what a student might do, whereas goals are written in future tense and based on what a person ought to be able to do. Competencies are developed based on the feedback and contribution from all stakeholders involved, that is, students, teachers, and community partners [9]. In the context of clinical training in midwifery, the stakeholders also include healthcare policy makers, midwifery curriculum developers and healthcare users. To meet the EU standards Polish medical universities offering midwifery degree courses are obligated to revise the curricula and introduce innovative solutions to improve the quality of clinical training. Mentoring is a strategy which could successfully contribute to increasing the effectiveness of clinical placements [3]. In 2017, a mentor preparation course for midwives was developed and conducted at the Medical University of Warsaw as part of a pilot program evaluating mentor-led clinical placements for midwifery students [3].

There is an increasing amount of evidence confirming the usefulness of mentoring in clinical training and studies have shown that it is well received by mentees [3, 14, 15]. Mentoring competencies and skills are recognized as essential for building successful relationships in many diverse areas such as business, education or research. The student-mentor relationship is crucial for the practice experience students are able to gain [16, 17]. The success of mentoring depends on adequate preparation, but not infrequently mentoring is introduced as an ad hoc exercise while it should be carefully planned and organized [18, 19]. The selection and matching of mentors/mentees alone is not a guarantee of success [19]. A scenario outlining the stages of the mentoring process must be developed and the implementation carefully planned [20]. Formal mentorship training of candidates preparing them to act as mentors is a key component [18–20]. Competencies for effective mentoring include ability to develop a stable, long-term relationship with the mentee, professional experience to share with them, good teaching skills and knowledge how to assess learning outcomes [20]. The experience of mentoring is increasingly perceived as reciprocal and teachers who act as mentors report improved productivity, work satisfaction and quality of life [21, 22]. In Poland, mentoring in the healthcare setting was initiated by the Jagiellonian University Medical College in Cracow in collaboration with Sheffield Hallam University (Sheffield, South Yorkshire, England). The two institutions developed an innovative Mentor Training Program for nurses [23]. In midwifery, however, mentoring as a formally recognized strategy for imparting clinical experience to either undergraduates or junior staff has taken longer to be adopted and it is still not commonly employed in Poland [3]. This may be partly due to the very common practice whereby experienced midwives, often informally referred as “mentors”, supervise and guide students or novice midwives who had no experience of the clinical situation [3]. The term “mentor” is often informally used to refer to experienced midwives supervising students or novice midwives [3]. Not infrequently such informal mentoring takes place during clinical placements. However, there is no officially recognized position of “midwifery mentor” in Polish hospitals and clinics and very few, if any, mentor preparation programs [3]. The aim of the project here described was to fill this gap, but it should be noted that a wider implementation of mentor-led clinical training in midwifery across Polish medical universities is subject to approval by the government recognized national accreditation body. With the growing role of mentoring confirmed by research, initiatives have been undertaken to develop and assess evaluate training programs specifically dedicated to healthcare mentors [18, 19, 21]. It is to be expected that formal implementation of mentoring as a component of training and professional development in midwifery would empower midwives and increase their professional competencies to act as autonomous healthcare workers as well as lead to more effective acquisition of clinical skills by mentees (midwifery students and novice midwives) contributing to better-quality maternity care [3, 24]. There is a paucity of empirical evidence on the actual effectiveness of different mentor preparation models and the methods of its evaluation [19]. The literature describes some strategies that should meet the expectations of mentors and mentees, but there are very few studies evaluating the specific needs of candidate midwifery mentors and their satisfaction with mentor preparation offered or actual implementation of mentoring in the hospital setting [25, 26]. The present study to some extent fills this gap in research as to the best of our knowledge the Mentor Training Program was the first mentor preparation course for midwives in Poland.

## Methods

### The reference to the model

In 2017, a mentor preparation course for midwives was developed at the Medical University of Warsaw as part of a pilot program evaluating mentor-led clinical placements for midwifery students. The Mentor Training Program (MTP) focused on presenting the concept of mentoring and its application in the clinical setting including such aspects as mentoring mindset, techniques and tools, developing of specific skills, and attainment of new competencies. It incorporated and modified some components used in other mentor-preparation programs.

### Pre-study preparation

Prior to the MTP initiation, its design, aims and content were presented in a guide available online to prospective participants. The workshop model was chosen because it allows participants to be actively engaged and was considered to aid in developing skills required to maintain the mentor/mentee relationship. All methods were employed in accordance with relevant guidelines and regulations in the Ethical Declarations.

### Setting

The Holy Family Specialist Hospital in Warsaw, 12–14 June 2017.

### Participants

Twenty-one midwives participated in the Mentor Training Program and comprised the study group. They were members of the obstetrics and gynecology teams at two hospitals in Warsaw (The Holy Family and Solec Hospitals). All participants had prior experience of involvement in midwifery clinical training. The characteristics of the study group are presented in Table 1.

**Table 1:** Characteristics of the study group (*n* = 21)

	n
**Age**	
≤ 25 years	2
26–36 years	10
37–45 years	4
≥ 46 years	5
**Academic level of qualification**	
Master’s level	11
Master’s level + specialty	9
Master’s level + teaching qualification	1
**Length of work experience**	
≤ 5 years	2
6–11 years	11
12–16 years	5
≥ 17 years	3
**Job title**	
Midwife	8
Midwife specialist	9
Midwifery coordinator	3
Maternity ward manager	1
**Place of work (hospital department)**	
Obstetrics	10
Gynecology	5
Pathology of pregnancy	1
Labor and delivery room	5
**Length of teaching experience**	
2 years	2
3–6 years	5
≥ 7 years	14

### Sampling

Potential participants had to meet the following criteria: at least 2 years full-time work experience, a master’s degree in midwifery, a teaching qualification or a completed specialty training and prior experience of teaching midwifery students on clinical placements. Candidates completed a questionnaire which included such items as the length of employment in the hospital department, job title, prior involvement in clinical training of midwifery students, their motivation to become a mentor and expectations related to the Mentor Training Program. The recruitment of 21 midwives who participated in the training and constituted the study group was carried out by the Program coordinator based on individual interviews and a thorough analysis of the questionnaires taking into consideration such aspects as interest in the program and motivation to participate, perceived personal strengths and weaknesses, expectations of the program and future midwife – student collaboration.

### Intervention

The recruited midwives took part in a Mentor Training Program. The training duration was 3 days. It consisted of 24 h of workshops (8 h/day) and focused on the concept of mentoring and the implementation of mentoring as a strategy for clinical training of midwifery students. The emphasis was on managing the skill acquisition process. The participants were encouraged to reflect on and evaluate what they learned and to share their opinions and experiences with others. The training activities used during the interactive workshops included multimedia presentations, breakout sessions, role playing and brainstorming in small groups (see Additional file 1: Annex 1 for the Agenda).

The course covered the following topics: the concept of mentoring in the context of clinical placement, key principles of mentoring program design, identification of mentees’ needs and setting measurable learning objectives, including the essential competency profile for midwifery students at a given stage in their course, creating a safe and supportive learning environment, effective methods of mentor-led clinical instruction, principles of mentee appraisal, benefits of mentoring in midwifery training and opportunities for actual implementation. The training was expected to provide the participants with a set of tools and techniques for effective mentoring. Upon completion of the training each participant received a certificate allowing them to provide mentor-led clinical training to midwifery students.

The Mentor Training Program and training course was developed and led by four Polish midwives who had previous experience in implementing mentoring programs within the Dedicated Education Units in Europe project (EC Project Number: 2015–1-BE02-KA202–012329) [27]. Within that project they attended a mentoring program and completed Dedicated Education Unit Mentorship Training for nurses/midwives and midwifery mentors conducted by trainers from the University College Leuven-Limburg at the University of Barcelona in September 2016.

### Data collection

This qualitative study was conducted in two stages and used the diagnostic survey method [28]. The study group received exclusively open-ended questions because we wanted the participants to answer the questions in their own words without undue interference or suggestions from the researcher. Each participant gave their written informed consent for inclusion in the study. The questionnaires were published online seven days before the training started and two weeks after its completion. In Stage 1, midwives accepted to participate completed a questionnaire consisting of 10 items. Six of the questionnaire items captured information about the participants (age, length of work experience, job description and prior experience of clinical teaching to midwifery students) and two questions, concerning the expectations of the Program and motivations to become a mentor, were open-ended (Table 2).

**Table 2:** Questionnaire used in Stage 1

1.	Age:
2	Length of work experience:
3	Place of work (hospital department):
4.	Length of current employment:
5.	Job title:
6.	Length of teaching experience: _______________ hours of clinical teaching; _______________Hospital/Department______year of study
7.	What motivates you to participate in the Mentor Training Program?
8.	What are your expectations of the Mentor Training Program?

In Stage 2, 2 weeks after their participation in the Mentor Training Program, the participants completed a questionnaire consisting of five open-ended questions (Table 3). The aim was to receive feedback on the program from the participants and to identify their further needs and unmet expectations.

**Table 3:** Questionnaire used in Stage 2

1.	What do you think of the Mentor Training Program?
2	What are the best aspects of the Mentor Training Program?
3	Do you see any weaknesses of the Mentor Training Program or any other issues related to it?
4.	Did the Mentor Training Program meet your expectations?
5.	What aspects of the Menor Training Program would you change?

### Data analysis

The answers were studied using qualitative content analysis by two researchers who were responsible for the coordination of the training and conducting the study, but were not involved in the actual training. Content analysis was used because it allows to identify the most important issues in the collected data and to compare the relative significance of the same issues across texts while data collection is unobtrusive and there is no researcher bias [29]. Eight research questions were selected, two questions from Stage 1 questionnaire and five questions from Stage 2 questionnaire (Table 4). The data was analyzed by the researchers and encoded (Midwife 1, Midwife 2, etc.). The same codes were used for the data from Stages 1 and 2 to perform a comparative analysis of answers given by the same person pre- and post- training.

**Table 4:** Research themes

**Stage 1**	
1. Motivations for participation in the Mentor Training Program	
2. Expectations of the Mentor Training Program	
**Stage 2**	
1. Opinions about of the Mentor Training Program	
2. The best aspects of the Mentor Training Program	
3. Weaknesses of the Mentor Training Program or any other issues related to it	
4. Expectations of own role in clinical training after completing the Mentor Training Program	
5. Proposed changes to the Mentor Training Program	

## Results

Twenty-one midwives from two teaching hospitals in Warsaw, Poland were recruited to participate in the Mentor Training Program. All of them completed the training and received certificates which allowed them to provide mentor-led clinical training to midwifery students. Given below are quotes from the participants’ answers to the questionnaires translated from the Polish language.

### Stage 1, conducted online seven days prior to the Mentor training program

1. What motivates you to participate in the Mentor Training Program?

All midwives who wanted to attend the MTP were interested in it because they knew little about mentoring and its uses in clinical training for midwifery students.

I am aware that clinical training for students needs to be changed and that is why I would like to know how I could use mentoring in my work. (Midwife 3)

The answers suggest that midwives as healthcare professionals do not have adequate knowledge of the benefits of mentoring as a tool for personal development and for this reason mentoring remains underutilized.

I like studying and learning more, continuing education is a must in my job. I don’t know what mentoring exactly is, but it seems something innovative and I’d like to learn more about it to move my career forward. (Midwife 15)

Some midwives, however, realized that in fact they already acted as informal mentors on clinical placements but did not know the theoretical foundations of a mentoring process and now wanted to know the principles for formal, effective mentoring.

I think that mentoring has been long an integral aspect of midwifery practice and if actually I am an informal mentor to students I would like to gain proper knowledge of what mentoring is. (Midwife 20)

I would like to know what I could do to become a better mentor. (Midwife 10).

The midwives were aware of the importance of proper mentor preparation and the need for relevant training.

I would like to be a true mentor but without proper preparation I wouldn’t feel comfortable in this role. (Midwife 3)

The Mentor Training Program is absolutely necessary to do your job well. (Midwife 2)

I would like to be a mentor, but I don’t know if I’d make a good one. (Midwife 21)

## What are your expectations of the Mentor training program?

The expectations were related mostly to the agenda. The participants wanted to learn how to prepare and implement a mentoring program in a hospital ward as there were no official guidelines on mentoring and mentoring methods in midwifery, including mentor preparation, a step-by-step guidance to starting and implementing a mentoring process or a question of mentor’s accountability.

The system of clinical training for midwifery students imposes upon us an obligation to follow innovative forms of instruction. I would like to learn how to prepare correctly and get started as a mentor. I want to become a professional mentor. (Midwife 15)

During the training I would like to learn how to conduct mentoring, to get to know what exactly you mean by a mentoring process. (Midwife 8)

One midwife expressed her concern about the lack of current standards for implementing mentoring in midwifery, e.g. Should a mentoring process be documented and how? Are there any limits of mentoring or appropriate boundaries of the mentoring relationship?

I hope that during the training I’ll be given instructions how to document mentoring of the student under my care. (Midwife 9)

The answers demonstrated that the midwives did not know who should be responsible for the mentor-led clinical training in their hospitals and what the key elements mentoring preparation and implementation should include.

I’d like to know how to organize mentoring in my workplace and how mentors and students are paired. (Midwife 8)

I hope that this training will be the first step to implement mentoring in midwifery and that such training will be continued in future. (Midwife 17)

### Stage 2, conducted online two weeks after the Mentor training program

1. What do you think of the Mentor Training Program?

The evaluation conducted after the training demonstrated a high level of satisfaction with the Mentor Training Program. The participants hoped that after completing the training they would be able to implement mentoring in their hospitals which would translate into better quality clinical training.

I’m really happy that I could attend such training. I hope that mentoring will become a permanent part of clinical training in midwifery. (Midwife 14)

Such training is really needed because if there are no mentors, mentoring won’t be possible and students will lose a lot. (Midwife 5)

The participants noted that mentor-preparation training was needed because many of their colleagues still saw the mentoring process as just selecting a mentor – student pair.

I learned a lot of interesting things about mentoring, especially about the principles of implementing a mentoring program. (Midwife 19)

Before I thought that mentoring is just pairing mentors and students. Now I know that mentoring is a huge process consisting of many stages. (Midwife 10)

According to many participants, the training changed their perception of the organization and delivery of the mentoring process. Before they did not know what actions to take to accomplish their mentoring successfully.

## What are the best aspects of the Mentor training program?

The training allowed the midwives to acquire new competencies and to further develop their teaching skills.

I think that the training made me a more confident mentor and I begin believing in my own potential. (Midwife 1)

The participants realized that to become a mentor one needed appropriate preparation and training.

Training courses are good for my personal development, especially when they teach me how to do things better. (Midwife 4)

The participants observed that the training they attended, the first mentor training program ever organized in Poland, might be an important step toward changing the current system of clinical training for midwifery students. They saw mentoring as having a key role in clinical placements in the future.

I think that such mentor training programs would facilitate implementing mentor-led clinical training of midwifery which would improve the quality of midwifery education. (Midwife 13)

One of the midwives noted that in the future the mentees could themselves become mentors and continue using mentoring as a strategy for personal and professional development. Another idea was for potential mentors to start as mentees.

I think it would be a good idea if a mentor could first be a mentee and observe their mentor and then become a mentor themselves. It could be an attractive proposition for younger people. (Midwife 17)

## Do you see any weaknesses of the program or any other issues related to it?

Although mentoring as a strategy for clinical training and support for nurses has been long used worldwide and its first implementation in Poland dates back to 2004, mentoring has not been yet adopted in the core clinical training curriculum for nursing and midwifery students. Two midwives expressed their concerns about the future of mentor-led clinical placements and chances of their implementation.

It’s been my first Mentor Training Program but I’m afraid it will take a long time to increase the number of qualified midwifery mentors. (Midwife 16)

Some midwives observed that because mentoring was not commonly known, mentors might be undervalued by their employers.

I’m worried that the role of a mentor is not appreciated enough by the employer. (Midwife 19)

Most perceived mentoring as an additional responsibility which could be time consuming and entail an additional burden.

I’d like to be a mentor, but I’m afraid it may result in extra time-consuming tasks. (Midwife 6)

The participants were aware that just completing the Mentor Training Program was not enough to conduct an effective mentoring program themselves. Many expressed their doubts whether they would cope as mentors and meet the mentees expectations and actual needs.

I don’t know if I’ll be able to offer mentees enough help to be considered by them an effective mentor. (Midwife 7)

## Did the Mentor training program meet your expectations?

The Mentor Training Program was found to be groundbreaking, valuable and meeting the participants’ expectations.

The Mentor Training Program is innovative and I’m happy to be part of it. (Midwife 12)

Most of the participants stressed the usefulness of the Program which focused on the specific application of mentoring to clinical training of midwifery students.

The training we had was very useful and I learned a lot. That was much needed training and I hope to use in my work with students what I learned during the sessions. (Midwife 18)

One midwife wrote that she could not really answer this question because before participating in the Program she did not know what she could expect.

I think, the answer is ‘yes’ but I really don’t know what else I could expect. No one has organized such training programs for midwives before. (Midwife 1)

## What aspects of this program would you change?

The participants observed that there was the continuing need to organize such training programs for both midwives who wanted to become mentors and those who already provided mentorship.

I’d like Mentor Training Programs to be cyclical because in case of doubts experienced in the mentoring process it would be good to have specialists to help you clear up these doubts. (Midwife 16)

One midwife stressed the need to improve the behavioral skills needed to become a mentor.

It was the first training of this kind; it might be a good idea to add more topics, e.g. strengthening of the mentoring skills. (Midwife 13)

The participants’ opinions confirm the continuing need for such training programs and their further development, refinement and expansion. More work is needed on the content and more topics should be covered. A comprehensive and long-term evaluation of the mentoring outcomes is required, ideally after the novice mentors have actually delivered a clinical mentoring program.

I’d like to share my experiences after actually running a clinical mentoring program. Workshops facilitate discussions and I’d like these meetings to continue. I want more. (Midwife 16)

## Discussion

Innovative teaching methods are required to improve the quality of clinical training for midwifery students and prepare them better for effective patient care in the future [14–17]. The use of mentoring appears to have a key impact on the training of nurses and midwives [3]. It is thought to contribute to the personal and professional development, improved acquisition of midwifery skills, building and maintaining networks in the workplace and career advancement [19]. To the best of our knowledge the Mentor Training Program for midwives described in this paper was the first initiative of this kind in Poland offering professional preparation for midwifery mentors. The subsequent feedback from the participants is evidence of its effectiveness. Published studies demonstrate that becoming a mentor increases job satisfaction and helps to prevent burnout [19–21]. The results of the present study confirm that midwives already involved in the clinical training of midwifery students were interested in the introduction of innovative teaching in clinical placements. Implementation of mentoring requires adequate preparation of the teaching and clinical staff to act as mentors. The candidates should undergo training presenting the concept of mentoring and its uses in specific areas as well as developing their mentoring skills [19, 22]. Although there are many studies describing actual mentor-preparation and mentoring programs, there is virtually no guidance on setting up a mentoring process. The results of this study show that pairing of mentors and mentees alone is not perceived by future mentors as a guarantee of success. The participants wanted instruction because mentoring as a strategy for midwifery training is still uncommon and they knew very little about it. The literature on mentoring reveals that there are virtually no effective methods to assess the knowledge, skills, personality traits and behaviors required of potential mentors [24, 25]. Not all midwives will be able to act as mentors although many seem to satisfy all formal requirements. It is assumed that a mentor training program could help them to reach their full personal and professional potential as well as to check whether they find this form of development attractive and satisfying [29, 30]. Although a specialist in a given field is sometimes described as a mentor, being a successful mentor requires in fact more than expert knowledge and skills [20]. Many of the midwives participating in the MTP felt that they already acted as informal clinical mentors but did not know whether their teaching and leadership skills were sufficient for engaging in a formal mentoring process. They believed that a mentor training could help them to increase their skills and develop as mentors. A number of studies, including a paper by Gray and Downer emphasized that mentors need to be provided with adequate preparation and support [31]. The midwives in the present study observed that a single training course was not enough to make one a mentor and they expressed the need for continuing dedicated structured education to become confident and competent mentors. The feedback we received from the MTP participants was encouraging but we are aware of the MTP limitations. The midwives volunteered to participate and therefore could be more motivated to learn about mentoring on the wards. The results also suggest the need to refine the MTP and to conduct further large-scale evaluation studies. Similarly, Johnson and Gandhi reported in their Mentor Training Program high participant satisfaction rates and a statistically significant improvement in the self-evaluated mentoring skills [19]. A number of authors point out that when creating a mentor training program for a particular professional group it may be necessary to eventually revise it to meet the specific expectations and needs of participants [19, 20, 24, 31]. It is important to continue supervision of the mentoring process run by the mentors as the MTP alone and its immediate evaluation do not predict the long-term distal outcomes for mentors and mentees. Ideally, programs to train effective mentors should include monitoring of clinical training led by the trained mentors, its outcomes for mentors and mentees, mentor satisfaction, and evaluation of mentors’ effectiveness from their own and mentees’ perspectives. Further research into the long term effects of mentor training is needed.

In many countries implementation of innovative solutions in education is considered a priority. The study reported in this paper demonstrates that in Poland mentoring is not commonly used and generally its value is underestimated by both academic teachers and healthcare managers responsible for clinical training [32]. The results of their research project reported by Moran and Banks demonstrate that mentors see themselves as essential to the delivery of midwifery programs but are not sure if student midwives or senior management are aware of their role and its value [21]. The lack of standards and generally poor knowledge of the mentoring process and mentor preparation, and the fact that the mentor’s role is usually underestimated in their workplace are responsible for the shortage of properly trained healthcare professionals who could act as mentors [24, 31]. As shown in the present study, its participants identified the above factors as the main obstacles hindering the adoption of mentoring as a strategy for clinical training in Polish medical universities.

Also, the awareness of the need to implement mentoring seems to be greater among nursing educators and in Poland professional nursing organizations have a longer history than midwifery associations. It seems necessary to focus on the needs of midwifery tutors willing to act as professionally trained mentors. Publishing the results of the present study may emphasize the need for a larger-scale structured mentor training for midwives not only in Poland, but also in other countries. International migration of midwives is recognized as a critical healthcare issue. Midwifery mentors trained through special mentor preparation programs could facilitate training of midwives from culturally diverse backgrounds and their adaptation to a culturally diverse workplace.

Papers by Gray and Downer and by Richmond raise a number of issues related to the delivery of a mentoring process. Setting the appropriate boundaries of the mentoring relationship may be difficult for novice mentors, mentors are required to set aside dedicated time and are additionally burdened with the task of writing reports evaluating the implementation and outcomes of mentoring [31, 33]. The participants of the MTP did not list such problems but they had no experience of mentorship and their perceptions could change when they established their first mentoring relationship. Papers evaluating mentor training programs show that structured Mentor Training Programs can improve mentoring skills if they are specifically tailored to meet the actual needs of the participants [19, 24, 31]. The midwives who attended the MTP assessed their preparedness to act as mentors as insufficient which indicates the need for providing further mentorship training focusing on the development of specific skills necessary for maintaining a successful mentor – mentee relationship, such communication skills, assertive mentoring, decision making and leadership.

## Conclusions

The Mentor Training Program for midwives, the first ever in Poland, was assessed by its participants as innovative, valuable and meeting their expectations although it should be noted that they all were determined to attend the training for which they volunteered. That strong motivation might have had an impact on their final opinion about the MTP The MTP presented the concept of mentoring and the role of mentors in the clinical teaching of student midwives. It increased the participating midwives’ awareness of the benefits of mentoring and showed them how to implement mentoring in midwifery practice. In the future, mentor training programs should focus on the development and reinforcing of these skills which are required to become a mentor. The response to the training confirmed the continuing need to organize such mentorship programs and revealed the areas which should be revised/developed to make the training relevant to the individual needs of the participants. Mentorship preparation programs need ongoing assessment of proximal and distal outcomes for mentors and mentees. The same applies to the use of mentoring in the clinical teaching for other healthcare professionals, which in Poland is in a pilot phase.

### Limitations of the study

The Mentor Training Program for midwives preceded a project implementing mentor-led clinical teaching for a small group of midwifery students and at that time there were no quantitative studies in this area. Implementation of the MTP and its subsequent evaluation using qualitative research methods can be seen as a limitation, but it allowed building and generating theories which can be further tested in quantitative studies. The number of participants (study subjects) was limited as this was a small-scale exploratory study but nevertheless a valuable source of knowledge revealed through individual perceptions, opinions and experiences.

We are planning to conduct Mentor Training Programs online to reach a larger number of midwives and to evaluate the level of satisfaction and the outcomes (self-assessed mentoring competencies) using standardized tools.

### Recommendation for practice

On 11 March 2020, the World Health Organization (WHO) Director General announced COVID-19 caused by the novel betacoronavirus SARS-CoV-2 as a pandemic [34]. As the COVID-19 pandemic spreads, at all levels of education there has been an increasing move from in-person classes towards teaching online which is hardly feasible in clinical training for midwifery students. Students lose several months of learning due to the shutdowns caused by COVID-19 [35, 36]. In this context, the role of the healthcare professionals as mentors to individual students gains new importance. Universities should implement risk management strategies to support students and help them cope with a variety of pandemic-related problems [37]. The COVID-19 pandemic has enforced changes to medical education and mentoring may meet these unprecedented challenges and serve the purposes of mediation and clinical practice facilitation as a valuable tool for increasing clinical knowledge and providing psychological support. More mentors and more mentor preparation programs are needed. The Mentor Training Program for midwives we developed can be conducted online which would allow training a larger number of midwifery tutors in a shorter time. It would require some modifications to respond to the challenges posed by the pandemic and include such aspects as providing psychological support to students and increasing the effectiveness of virtual midwifery teaching. Eventually, e-mentoring programs may evolve as an innovative approach to clinical training support to midwifery students.

## Supplementary Information

**Additional file 1: Annex 1**. The agenda of the Mentoring Training Program (MTP).

**Additional file 2: Annex 2**. Active observation and critical reflection journal.

## Data Availability

All data generated or analyzed during this study are included in this published article.

## Abbreviations

CBE: Competency-based education
EU: European union
MTP: Mentor training program
WHO: World health organization
COVID-19: Coronavirus disease 2019
