# Supplementary material for: Evaluation of a Mentor training program for midwives in two hospitals in Warsaw, Poland - a qualitative descriptive study
Source: BMC Med Educ. 2021 Jun 15;21:345. doi: 10.1186/s12909-021-02769-7 (PMC8204609; doi:10.1186/s12909-021-02769-7)
Supplement: Supplementary file 1 — Additional file 1: Annex 1. The agenda of the Mentoring Training Program (MTP). [file 12909_2021_2769_MOESM1_ESM.docx]

**Annex 1**

The agenda of the Mentoring Training Program (MTP)

| **Day 1**  Training time: 8 hours  Concepts and theories of mentoring | | | | |
| --- | --- | --- | --- | --- |
| **Session/length** | **Theme** | **Teaching method** | **Teaching activity** | **Evaluation of effectiveness** |
| **INRODUCTION**  10 min | 1. Opening remarks 2. Training program overview, the main objective of the training and expected outcomes 3. Day 1 agenda | Introductory talk | Oral presentation  Training agenda | Concentration  Motivation |
| **MAIN SESSION**  50 min | - - - 1. Mentoring: the definition and origin (concept, aims, models)       2. Mentoring as a strategy for personalized learning - Individual learning pathway - Wygotowski zone of proximal development   - - 1. Models of mentoring (Clutterbuck mentoring, Dedicated Education Unit, Manchester Gold). | Lecture | Multimedia presentation | Concentration |
| **Break** 10 min | | | | |
| **SESSION CONTINUED**  120 min | Mentoring and organizational learning  Learning through experience (the Kolb Learning Cycle), Competency-based education theory (CBE)  Effective communication (active listening, barriers, feedback)  The Ofman Core Quality Quadrant Model | Lecture  Discussion | Multimedia presentation | Concentration  Engagement in discussion |
| **Break**  40 min | | | | |
| **WORKSHOP**  **120 min** | 1. Principles of effective feedback. 2. Developing critical and reflective thinking skills (theory, models, reflectivity). 3. Setting goals (SMART, planning). | Discussion  Metaplan technique | Activities in groups | Activity  Interest |
| **Break**  10 min | | | | |
| **FINAL SESSION OF THE DAY**  **120 min** | 1. Starting an active observation and critical reflection journal 2. Participants’ reflections on Day 1 of the training | Practical teaching/learning methods  Content-based exercises  Project method  Discussion | Individual work  (Annex 2) | Completing the active observation and critical reflection journal  Activity |

| **Day 2**  Training time: 8 hours  Mentoring as an innovative form of practical training | | | | |
| --- | --- | --- | --- | --- |
| **Session/length** | **Theme** | **Teaching method** | **Teaching activity** | **Evaluation of effectiveness** |
| **INTRODUCTORY SESSION**   1. **min** | 1. Day 2 agenda | Introductory talk | Oral presentation  Training agenda | Concentration  Motivation |
| **MAIN SESSION**  **120 min** | 1. Mentoring in midwifery  - Mentoring around the world - Mentoring in Poland  1. Experiences in mentoring for clinical training of midwifery students 2. Roles and tasks of mentors and mentees, mentor competency profile | Lecture | Multimedia presentation | Concentration |
| **Break**  10 min | | | | |
| **WORKSHOP**  **120 min** | 1. Preparing students for mentor-led clinical training 2. Criteria for evaluating student work, progress and achievements, including clinical skills attained 3. Analysis of the criteria for the evaluation of clinical skills of midwifery students. | Discussion  Metaplan technique | Exercises in groups | Activity  Interest |
| **Break**  40 min | | | | |
| **CONTINUATION**  **120 min** | 1. Advantages of mentoring in the clinical training of midwifery students 2. Opportunities for implementing mentoring in clinical teaching | Lecture | Multimedia presentation | Interest  Engagement |
| **Break**  10 min | | | | |
| **FINAL SESSION OF THE DAY**  **110 min** | 1. Creating a mentor competency profile 2. Participants’ reflections on Day 2 of the training | Practical teaching/learning methods  Content-based exercises  Project method  Discussion | Exercises in groups (Appendix 2) | Activity  Engagement  Completing the active observation and critical reflection journal |

| **Day 3**  Training time: 8 hours  Mentoring in the clinical training of midwifery students | | | | |
| --- | --- | --- | --- | --- |
| **Session/length** | **Theme** | **Teaching method** | **Teaching/learning activity** | **Evaluation of effectiveness** |
| **INTRODUCTORY SESSION**  10 min | 1. Day 3 agenda 2. The main objective of the training program | Introductory talk | Oral presentation  Training agenda | Concentration  Motivation |
| **MAIN SESSION**  50 min | 1. Clinical training curriculum for midwifery students  - Potential uses of mentoring for teaching basic maternity care and specialist care during pregnancy and labor | Lecture | Multimedia presentation | Concentration |
| **Break** 10 min | | | | |
| **WORKSHOP**  **120 min** | 1. Stages of the mentoring process  - Developing a mentoring relationship - Setting goals - Ending a mentoring relationship - Further activities | Discussion  Metaplan technique | Exercises in groups | Activity  Interest |
| **Break**  40 min | | | | |
| **WORKSHOP**  **120 min** | 1. Implementing a mentoring program: organizing a mentor-led clinical training for midwifery students | Practical teaching/learning methods  Project method | Group work (Appendix 2) | Activity  Interest  Engagement |
| **Break**  10 min | | | | |
| **FINAL SESSION**  **120 min** | 1. Participants’ reflections on Day 3 of the training Participants’ reflections and observations based on their journals | Discussion | Exercises in groups | Activity  Engagement  Writing the active observation and critical reflection journal |
