# Supplementary material for: Evaluation of a Mentor training program for midwives in two hospitals in Warsaw, Poland - a qualitative descriptive study
Source: BMC Med Educ. 2021 Jun 15;21:345. doi: 10.1186/s12909-021-02769-7 (PMC8204609; doi:10.1186/s12909-021-02769-7)
Supplement: Supplementary file 2 — Additional file 2: Annex 2. Active observation and critical reflection journal. [file 12909_2021_2769_MOESM2_ESM.docx]

**Annex 2. ACTIVE OBSERVATION AND CRITICAL REFLECTION JOURNAL**

ACTIVE OBSERVATION AND CRITICAL REFLECTION JOURNAL is one didactic tool used within the Mentor Training Program. Recording of observations and impressions in each day of the MPT allowed the participants to reflect on the perceived training-related problems, needs and expectations arising during the workshops.

The journal guides the writer through the reflective thinking cycle concerning the innovative forms of personalized practical education to improve the quality of practical clinical education.

Exercise for the participants

| **Day 1**  Concepts and theories of mentoring | |
| --- | --- |
|  | What do you think in general about Day 1 of the training? |
|  | What did you find out/learn during the sessions? |
|  | What was interesting/inspiring? |
|  | Create a Personal Development Plan for yourself. Set SMART goals:  **S** – specific  **M** – measurable  **A** – achievable  **R** – realistic  **T** – timely defined |

| **Day 2**  Mentoring as an innovative form of practical training | |
| --- | --- |
|  | What do you think in general about Day 2 of the training? |
|  | What did you find out/learn during the sessions? |
|  | What was interesting/inspiring? |
|  | Create your competency profile. |

| **Day 3**  Mentoring in the clinical training of midwifery students | |
| --- | --- |
|  | What do you think in general about Day 3 of the training? |
|  | What did you find out/learn during the sessions? |
|  | What was interesting/inspiring? |
|  | Mentoring in practice: design a mentoring program |

Below are 12 questions the MTP participants were requested to answer after the training. The feedback allowed the trainers to perform a preliminary evaluation of the MTP usefulness.

| **RESUME** |
| --- |
| Answer the following questions: |
| 1. What are the stages of mentoring? 2. Do you think it is possible to implement mentoring in the clinical training of midwifery students? 3. What are the problems related to the implementation of mentoring in the clinical training of midwifery students? 4. Who should teach clinical skills? 5. Who should assess the clinical skills the student acquired? 6. Why should a midwifery student be assessed in practice setting? 7. What documentation of mentor-led clinical training do mentors and mentees need to provide? 8. Is a mentee required to work with their mentor all the time? 9. What should be done when a mentoring relationship fails? 10. How to help a mentee who has problems with the acquisition of the curriculum -required competencies and skills? 11. How to reward mentees for achievements? 12. What to do in case a mentee does not agree with their mentor’s opinion and assessment? |
